# Forensic human image identification using medical indicators

**DOI:** 10.1080/20961790.2020.1838252

**Published:** 2021-01-08

**Authors:** Jinhua Zeng, Xiulian Qiu, Shaopei Shi, Xinwei Bian

**Affiliations:** Shanghai Key Laboratory of Forensic Medicine, Shanghai Forensic Service Platform, Academy of Forensic Science, Ministry of Justice, Shanghai, China

**Keywords:** Forensic sciences, medical indicators, forensic identification of human images, medical diseases

## Abstract

Diseases not only bring troubles to people’s body functions and mind but also influence the appearances and behaviours of human beings. Similarly, we can analyse the diseases from people’s appearances and behaviours and use the personal medical history for human identification. In this article, medical indicators presented in abnormal changes of human appearances and behaviours caused by physiological or psychological diseases were introduced, and were applied in the field of forensic identification of human images, which we called medical forensic identification of human images (mFIHI). The proposed method analysed the people’s medical signs by studying the appearance and behaviour characteristics depicted in images or videos, and made a comparative examination between the medical indicators of the questioned human images and the corresponding signs or medical history of suspects. Through a conformity and difference analysis on medical indicators and their indicated diseases, it would provide an important information for human identification from images or videos. A case study was carried out to demonstrate and verify the feasibility of the proposed method of mFIHI, and our results showed that it would be important contents and angles for forensic expert manual examination in forensic human image identification.

## Introduction

Disease is a nightmare that mankind has always wanted to get rid of, which broadly refers to any condition that affects the normal appearances and functioning of the body and are associated with specific symptoms and signs. The categories of diseases mainly consist of the mental, the physical, the genetic, the emotional and behavioural and the functional ones.

On the one hand, when facing continuing illnesses or chronic diseases, the individual has to modify or adapt behaviours to accommodate current health status, such as walking patterns. It is commonly supposed that the illness can exert influences on appearances, behaviours and mentality of peoples. On the other hand, certain appearance and behaviour traits can reveal vital clues to underlying diseases. Visual cues allow us to recognize illness from wellness. In the traditional Chinese medicine, one of the four standard diagnosis methods is looking, in which the inspection is focused on the patient’s physical appearance and behaviour. The evidence of physical changes as a consequence of abnormal conditions will be identifiable with certain telltale signs. Here, some common diseases were introduced which could affect appearances and behaviours with apparent medical indicators.

### Adenoid hypertrophy

**Figure 1.**
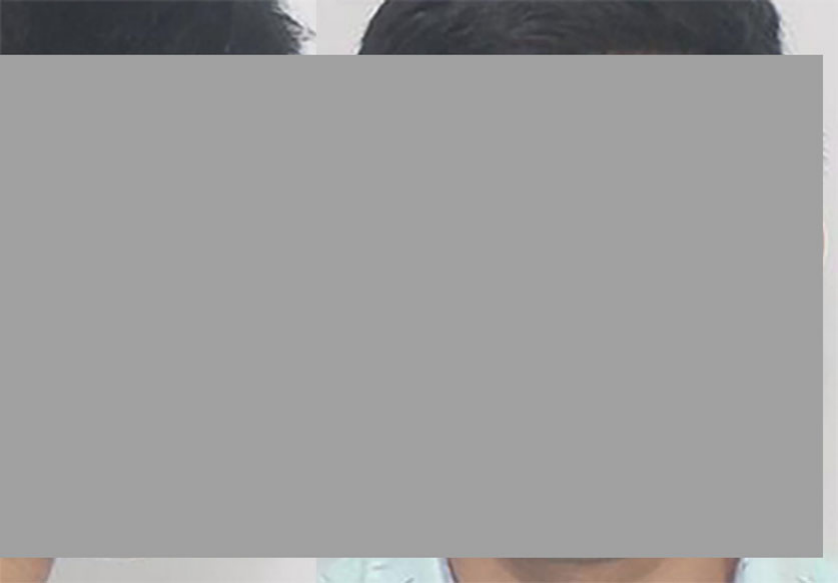
The typical facial features of the adenoid facies.

Adenoid hypertrophy is the unusual growth of the adenoid first described by Wilhelm Meyer in 1868, which will cause an obstruction of the nasal airways and result in mouth breathing. Nasorespiratory function is supposed to exert a dramatic effect upon craniofacial morphology [1]. The long-term adenoid hypertrophy in the period of the active growth will cause altered tongue and mandibular positions and finally develop into a dentofacial growth anomaly defined as “adenoid facies”. People with “adenoid facies” typically have a vertically long lower third facial height, narrow alar bases, lip incompetence, a long and narrow maxillary arch and a greater-than-normal mandibular plane angle [1–3]. In addition, a person with mouth breathing induced by the adenoid hypertrophy may raise one's head higher and then cause a risk for head posture deviation [4]. People with adenoid facies are generally characterized by an open mouth posture, an incompetent lip seal, a short upper lip, a poorly developed nose with small nostrils, increased anterior face height, a narrow upper dental arch, a steep mandibular plane angle and a retrognathic mandible [1–3,5,6]. The details can be referred to the literature [5], and a typical adenoid face was shown in Figure 1.

### Facial asymmetry

**Figure 2.**
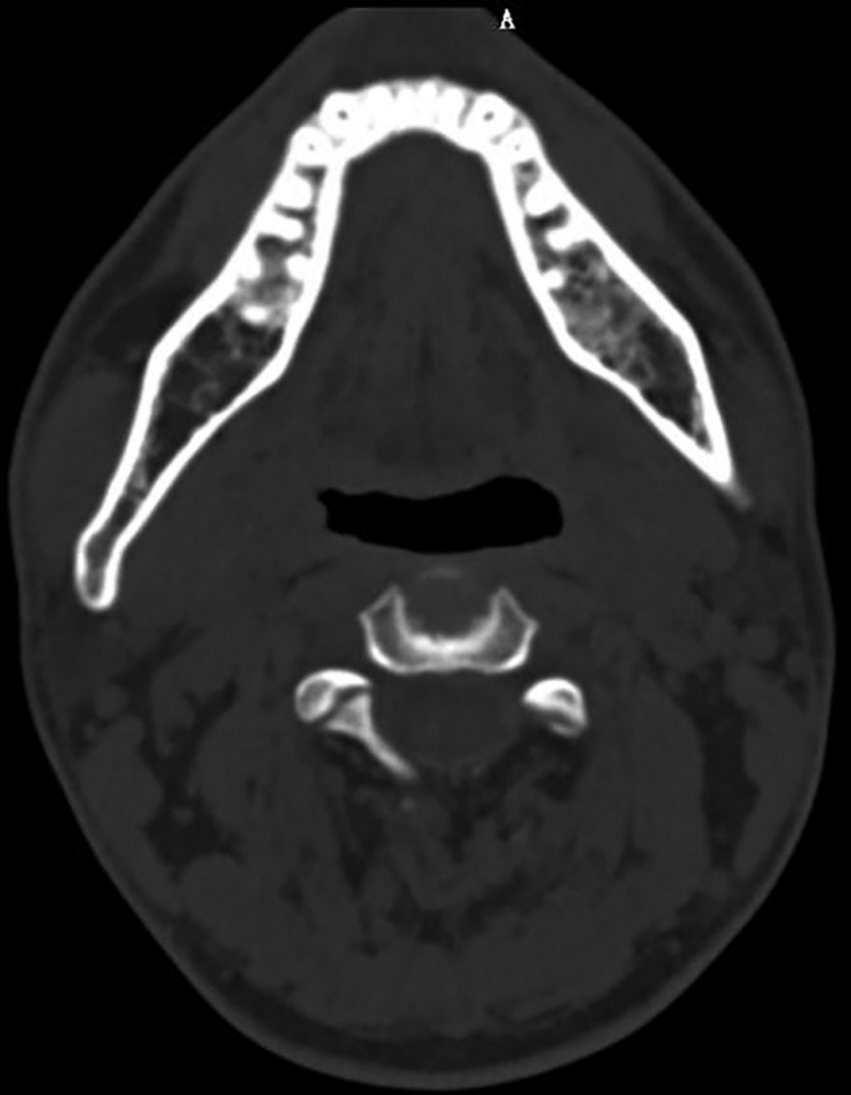
One example of face asymmetry by CT scanning.

Facial asymmetry is highly prevalent in the overall population. Almost all people have some degree of asymmetry on their faces and they are often presented subclinically. However, significant facial asymmetry may cause both functional and aesthetic problems. Facial asymmetry can be attributed to three main causes: (1) congenital, originating prenatally; (2) acquired, resulting from injury or disease; and (3) developmental, arising during development under unknown aetiology [6,7]. The congenital conditions consist of the facial clefts, hemifacial microsomia, neurofibromatosis, congenital muscular torticollis and so forth. Acquired changes associated with facial asymmetry comprise hyperplasia or hypoplasia of the condyle, ankylosis of the temporomandibular joint, trauma, fracture and so on. The developmental factors of asymmetry remain unknown in many cases. The habitual mastication on one side, constant facial pressure during sleep exclusively on one side, deleterious oral habits or unilateral crossbite as being some of the causes of disharmony have been reported [8,9]. In addition, facial asymmetry is observed more frequently in the lower face than the upper face [10]. One example of face asymmetry was shown in Figure 2.

### Gait disorders

Walking is one of the most common activities during daily living. However, it depends on a complex interplay of the nervous, musculoskeletal and cardiorespiratory systems. In order to have a normal gait, the locomotor function, balance, postural reflexes, sensory function and sensorimotor integration, motor control, the musculoskeletal apparatus and cardiopulmonary functions should be intact [11].

The personal gait is strongly affected by the factors comprising age, personality, mood and sociocultural environment, and it is a sensitive indicator of health status. Moreover, the diseases-related gait disorders are relatively common in each level of ages, and significantly more observed in the elderly [12]. The main causes of gait disorders [11] can be classified into neurological conditions (e.g. sensory or motor impairments), orthopaedic problems (e.g. osteoarthritis and skeletal deformities) and medical conditions (e.g. heart failure, peripheral artery disease [13], respiratory insufficiency). A phenomenological classification of common gait disorders consists of hemispastic gait with characteristics of unilateral extension and circumduction, dystonic gait with abnormal posture of foot/leg, waddling gait with broad-based, swaying, drop of swinging leg, and so on [11,14].

### Substance abuse

Substance abuse is a worldwide issue continuing to plague people of all walks of life, which is one of the primary health and social concerns in current world. Substance abuse has the deteriorating effect on mental health [15]. Meanwhile, it can also cause physical effects to addicts [16]. Drugs can change physical appearance both in faces and bodies.

For example, heroin can cause severe weight loss and skin sores. Steroids can cause baldness. The accelerated aging was observed in the addiction of opiates [16]. Continued abuse of methamphetamine would result in skin sores and “meth mouth” [17] with rotten teeth, and other mouth hygiene diseases.

**Figure 3.**
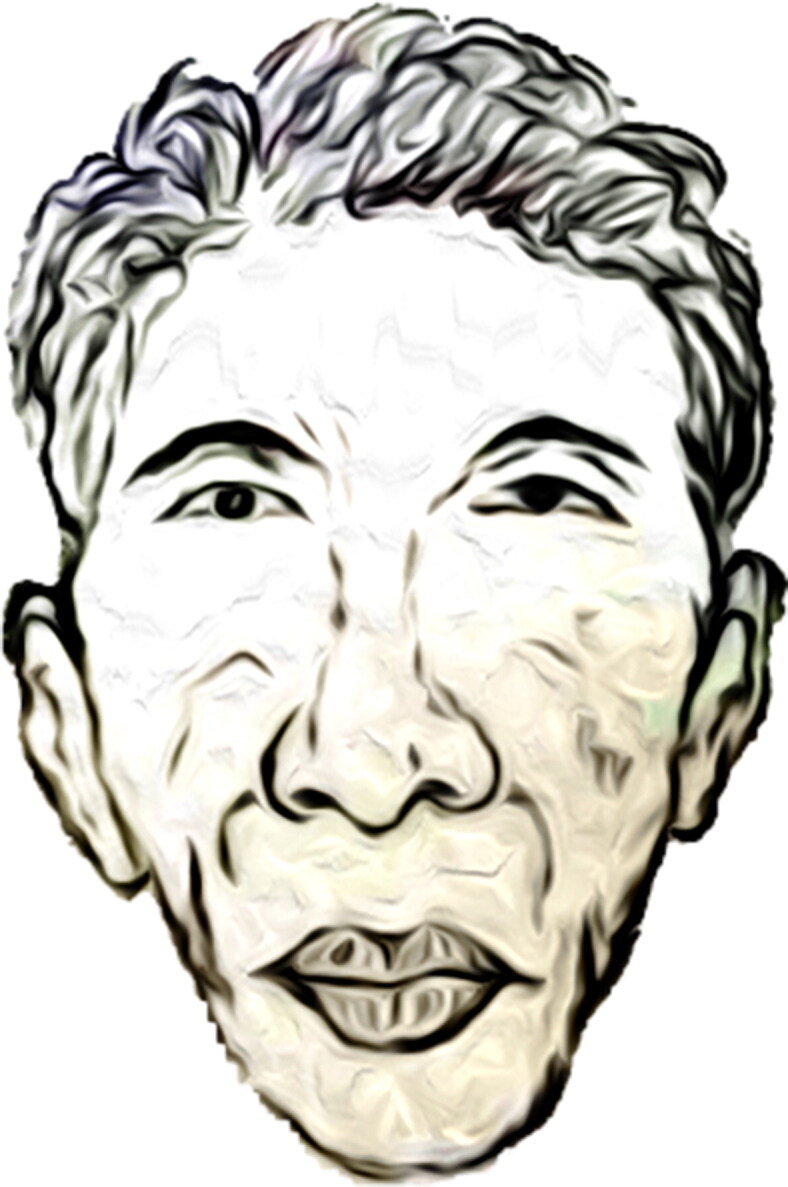
The sketch of a typical acromegaly face.

With regard to noticeable variations in faces after drug abuse, the most common appearance signs are dull skin, acne breakouts, bloodshot eyes and potential self-inflicted wounds. In addition, Some literature [18,19] is specialized in the face recognition studies considering the variations presented in the facial appearance with prolonged substance abuse.

The above medical signs caused by substance abuse observed in the human images of criminal offenders can be verified with the drug possession history of suspects.

### Acromegaly

Acromegaly is a chronic disease that results from excessive secretion of growth hormone after the growth plates have closed [20], and it usually happens with somatotroph adenomas [21].

The statistically significant changes of the facial skeleton were found in the lower jaw with its elongation given by the chin prominence and the condyle [22]. The facial features of acromegaly are typical with the characteristics of widening teeth spacing, prognathism, frontal-bone enlargement, nose enlargement, zygomatic arch prominence, brow ridge and forehead protrusion or prominence, soft tissue swelling (lips, nose, ears enlargement) and skin thickening [21]. In addition, the excessive facial-tissues would result in deep nasolabial-folds, enlarged noses and brows. The pathological changes in face and skull in acromegaly are ones of the characteristic clinical presentation [23]. One of the sketches of an acromegaly face was shown in Figure 3.

**Figure 4.**
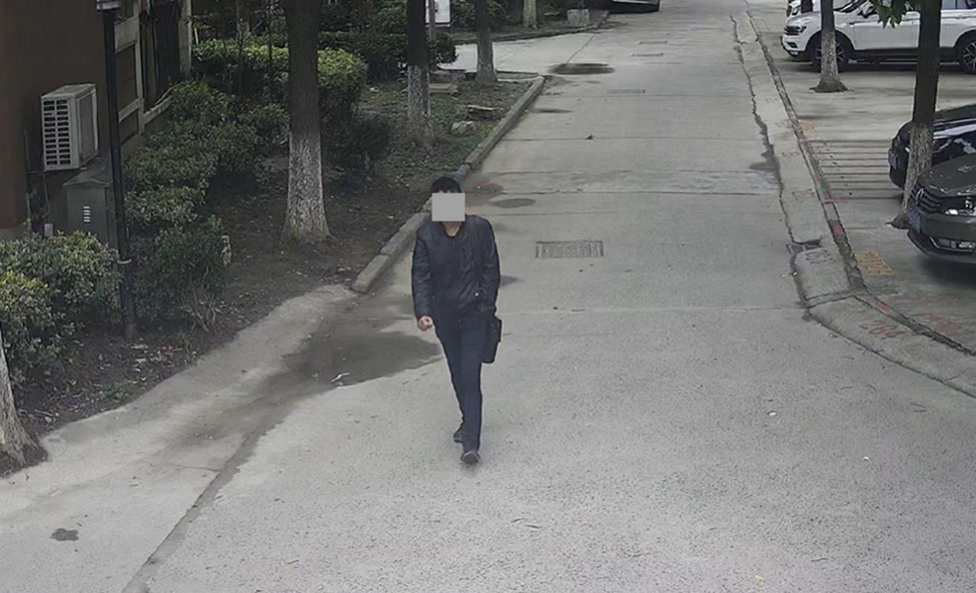
A screenshot of a video in the crime scene. The upper part of the face is blurred for privacy.

Some research was carried out to recognize acromegaly by directly using face recognition technology [21,24,25]. Automatic detection of acromegaly has been possible from acromegaly face recognition to screen in clinical settings.

### Other diseases

There are plenty of diseases that can affect the appearance and function of human beings apart from the aforementioned ones. For example, the characteristic faces of patients with 22q11.2 deletion syndrome [26,27] comprise vertically long faces, narrow palpebral fissures, fleshy nose with a broad nasal root and so forth. And children with obstructive sleep apnoea have similar craniofacial characteristics as those with adenoid hypertrophy [28,29]. Human characteristics depicted in images or videos, such as medical characteristic faces [30] and abnormal gaits, can be served as important indicators of diseases. They can be used in the field of forensic identification from photographs.

We introduced the medical indicators as mentioned above to the field of forensic identification of human images (FIHI), which we called medical forensic identification of human images (mFIHI). In the mFIHI, the human image features comprised the medical indicators, in addition to the traditional human features in the FIHI. In the examination, the medical indicators of the questioned human images were extracted from perpetrators’ appearances and behaviours in videos. These videos might be from the commission of their crimes or their travels to and from their crimes. The corresponding human features were checked out from the known human images or from the medical history of criminal suspects. The conformities and differences of the medical indicators between the questioned human images and the known human images were evaluated to assist the final opinions of FIHI. We further classified the categories of human image features into the meta features (MFs) and the domain features (DFs) in the FIHI.

### The MFs of human image

The MFs are the most intuitive, concise, neutral meanings of human image characteristics, and they are the most basic features in the FIHI. Traditional human features could be classified into these types, such as the head and facial morphological features, facial configuration relationship features and so forth. Of course, some MFs could be further classified and refined in hierarchies. For instance, the facial features could be divided into the morphological features of eyes, noses, mouths and so forth. Then, the eye features could be further divided into holistic eye shape features, upper eyelid features, under-eye bag features and so forth. The holistic eye shape features were further classified as round eyes, triangular eyes and so forth.

### The DFs of human image

The DFs is through the “domain encapsulation” of the MFs with the professional knowledge in specific fields. They are typically composite features consisting of a specific subset of the MFs and can be seen as an extension, optimization, upgrading and sublimation from the MFs. The medical indicators studied in this article were one of the typical DFs with the professional knowledge in medicine. The categories of the DFs could also be classified into static and dynamic features, such as the features of medical characteristic faces [30] and gait disorders in the mFIHI. The DFs features are more abstract than the MFs, and more reliable and stable as well. What is more, they are more robust to the varieties of imaging conditions, such as image artifacts, pose angles, perspective and image quality. However, some MFs are susceptible to changing conditions as a result of aging, expression changes, poor imaging quality and so on. For example, the subtle human image features, such as moles and scars, could only be observed in human images with high image quality.

## Case study

A case study was carried out to validate the proposed method. In a criminal case of stealing two-wheeled electric cars, the thief rode the stolen car out from the gate of a housing estate. A surveillance video recorded the images of the thief, as shown in Figure 4.

**Figure 5.**
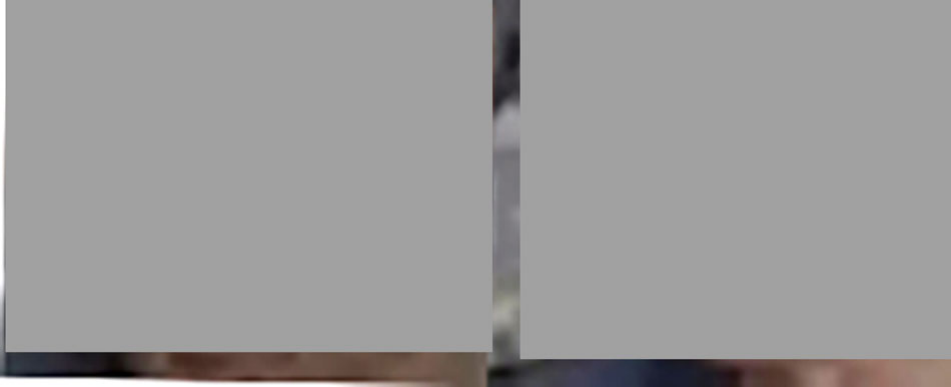
The facial images of the criminal. Only lower part of the face was presented for privacy.

After the initial investigation, a suspect was locked and arrested. However, the suspect was tight-lipped about the case and refused to tell the truth. There was no other available evidence in this case except for the identity of the criminal depicted in videos. So the author were designated as a forensic expert to examine the identity of the subject depicted in the video. The apparent feature of the criminal was that his mouth was always in the “open” state in videos. The causes of this open mouse posture could exclude the behaviours such as speaking, eating that need opening his mouth persistently.

In the case, the thief’s facial appearance and behaviour were fit to the medical indicators of the adenoid facies, which are characterized by an open mouth posture, a short and upturned upper lip, increased anterior face height, mandibular retrognathism, as shown in Figure 5.

**Figure 6.**
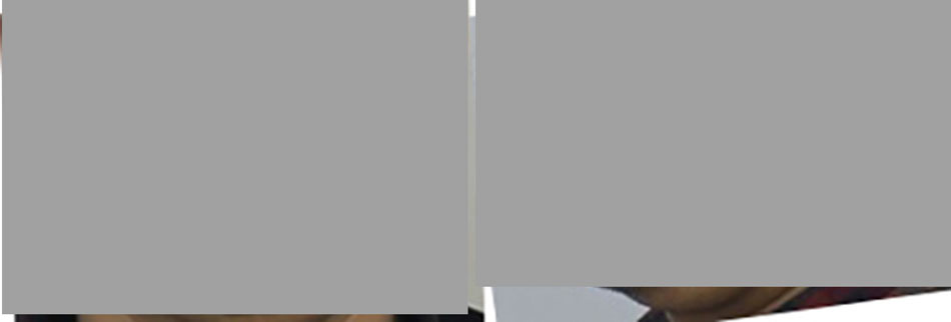
The lower part of the suspect’s face.

**Figure 7.**
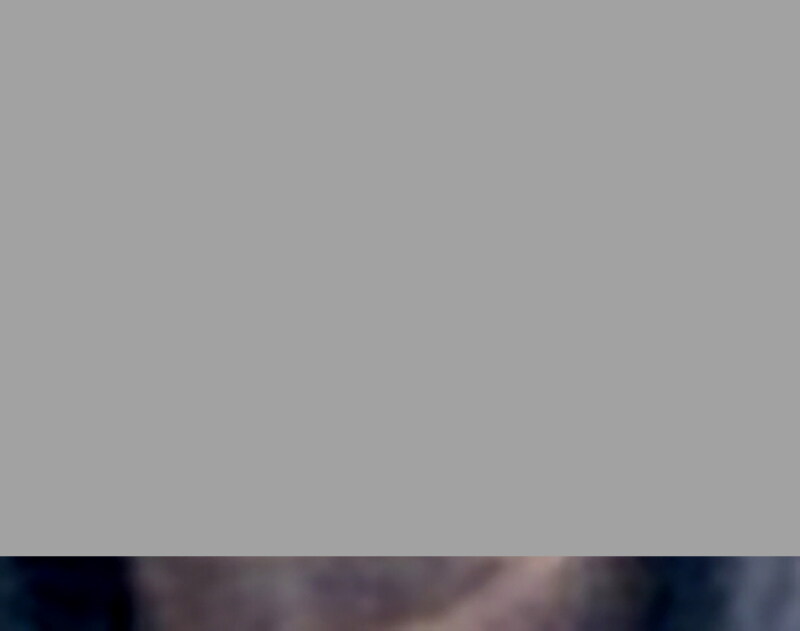
The lower part of the suspect’s face in different cases.

After the morphological feature examination of the questioned human images of the criminal, we further analysed the corresponding medical indicators of the known human images of the suspect. For privacy policy, only lower part of the suspect’s face was presented here, as shown in Figure 6. It could be found that the suspect’s face was a typical adenoid facies with characteristic extra oral appearance of the facial features, that was, an open mouth posture, an incompetent lip seal, a short upper lip, a steep mandibular plane angle and a retrognathic mandible. In addition, the suspect was a repeat offender, and his open mouth posture was stably observed from the frontal facial images in several cases, such as the face shown in Figure 7. The medical indicators of the adenoid facies had been both depicted in the questioned and the known facial images, i.e., the human images in crime scences and the supect images, respectively. which could be used as important medical domain features for FIHI.

FIHI is to compare images of people to provide identification evidence to courts or for police investigations. Its history can be extended back for many decades. In the examination, forensic experts make judgments of whether the questioned human images in images or videos and the known ones are the same person. In order to give reasonable expert opinions, human features revealed in images or videos should be comprehensively inspected, which consist of head morphological features, facial components and their spatial relationships, beard features, wrinkle features, body silhouette features, special human marking features, human dress and adornment features and other human dynamic features, such as gaits. The general categories of the human features in images can also be divided into static features and dynamic features. The most common ways of forensic identification of people from images is through face comparisons by means of holistic comparison, morphological analysis, photo-anthropometry and superimposition [32,33]. Compared with the traditional human image features for examination, the medical indicators would act as a valuable source for human image feature comparison. The underlying basis of the medical indicators is their associated diseases. In the case, through the examination of the head morphological features, we found many distinct MFs of the criminal in the videos. By combining the knowledge in the medical field, we found that the combination of the detected MFs can support the hypothesis that the criminal has the medical indicators of the adenoid facies. Then, the related diseases which can cause the adenoid facies are considered in the examination of FIHI by experts. Therefore, we concluded that medical indicators can be used as important human features for human identity authentication in the FIHI.

When using the medical indicators in the practical cases, it is important to note that the diseases have their own priorities, such as the different severity. There is no obvious medical signs with regard to some mild diseases. In addition, in the practical case examination, the acquisition of the medical history of suspects will be very useful since it provides the intuitive information of the diseases and lays the factual foundation for following comparison examination of the medical indicators. The medical examination of suspects in the current workflow of polices may offer a feasible way to preliminarily obtain the health status of suspects. In future, some specially targeted medical examination items can be added into the physical examination which will provide the important clues for the examination in the mFIHI.

It should be concerned how to evaluate the value of the medical indicators. The feature value evaluation is important in the FIHI. The general principles consist of the facts that value weight of rare features is higher than common observed features, detail features are more important than outline features, and the stable characteristic value weight is higher than the variable ones and so forth. When facing MFs and DFs of human images, we think that the latter is more valuable. For example, in our studied case, the medical indicators of the adenoid facies are more important than the simple additive summation of single MFs, such as a short upper lip, a steep mandibular plane angle and so forth. The DFs are the integration of the knowledge in special fields and the MFs information, which are the domain knowledge encapsulation and can be seen as an optimization and sublimation from their constituted MFs.

Before using the domain human image features, forensic experts should be trained to acquire the related acknowledge in specific fields, such as the medical characteristic face of the adenoid facies in our case study. We hope our proposed mFIHI will contribute to the small but growing body of emerging technology in FIHI, extending the contents and angles for human image examination, which can be finally incorporated into training, proficiency standards, competency and best practices.

## Conclusion

In this case, we offered our expert opinion that the questioned and the known human images were from the same person, which was accepted by court. The medical indicators are valuable features for forensic human image identification; however, it does not mean the forensic opinion is formed by using these features alone. In the case, the meta human image features had been comprehensively examined and compared. For example, there was a black mole in the lower right nostril of the suspect, and a black spot was observed in the same location. However, the domain human image features in medicine made their distinctive contribution to the final expert opinion.

## Note

Figure 1, 5, 6 and 7 are covered for ethical concerns.
